# A rare complication of pulmonary tuberculosis - Pulmonary venous thrombosis with atrial extension

**DOI:** 10.15171/jcvtr.2017.09

**Published:** 2017-03-18

**Authors:** Onkar Balasaheb Auti, Ranganath R, Murali Mohan BV, Vimal Raj

**Affiliations:** ^1^Department of Radio-diagnosis, Narayana Health City, Bangalore, India; ^2^Department of Pulmonology, Narayana Health City, Bangalore, India

**Keywords:** Pulmonary Venous Thrombosis, Tuberculosis, Complications of Pulmonary TB

## Abstract

Pulmonary tuberculosis (TB) is serious public health problem in India and worldwide. The mortality rate in pulmonary TB is high in those with advanced disease and in presence of complications. It presents with wide variety of complications of which haematological complications are rare. Many cases were reported pulmonary TB associated with deep venous thrombosis, hepatic and portal venous thrombosis, central retinal venous thrombosis and sagittal sinus thrombosis in TB meningitis. We report a rare case of pulmonary TB with pulmonary venous thrombosis and atrial extension. To our best knowledge, this is the first case reported of its kind.

## Introduction

Pulmonary tuberculosis (TB) shares great disease burden globally as well in India. Mortality in pulmonary TB cases is high due to advanced disease and complications. Early diagnosis and starting proper treatment in complicated pulmonary TB is of great importance in terms of patient recovery. TB can cause wide variety of complications, however hematological manifestations are rare. Many cases reported with tuberculosis and occurrence of deep venous thrombosis.^1-5^ Other haematological complications of pulmonary TB were also reported in literature like thrombosis of hepatic, portal, retinal veins etc.^6-10^ We present a case of pulmonary tuberculosis causing pulmonary vein thrombosis with atrial extension. To our best knowledge, it is the first case reported of its kind.

## Case report

A 35-year-old female presented to the chest physician with history of fever and breathlessness since 3 months associated with cough and expectoration. Symptoms aggravated since one week. She also gave history of pulmonary tuberculosis and was undergoing the treatment for the same (ATT category 2) since one month. She gave the history of previous surgery for psoas abscess. On examination, her pulse and blood pressure were stable. She was found to be in respiratory distress and there was evidence of right lower lobar pneumonia. Chest x ray showed right lower lobar collapse. Echo showed presence of mobile mass in roof of left atrium (LA) extending into mitral annulus. Mild mitral regurgitation (MR) was also noted.

**Figure 1:**
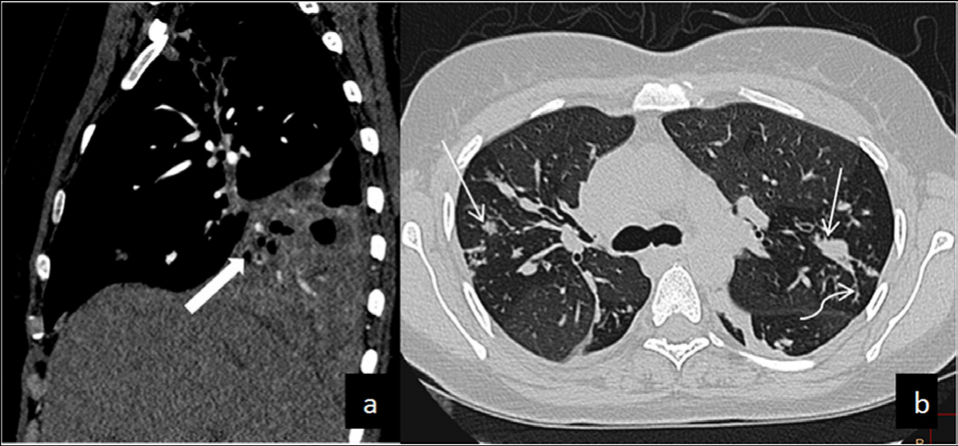
(a) Sagital CECT images showing consolidation of right lower lobe with cavitations (block arrow). (b) Axial MDCT in lung window showing multiple nodules in bilateral upper lobes (straight arrows). Note the tree in bud appearance in posterior segment of left upper lobe (curved arrow).

**Figure 2:**
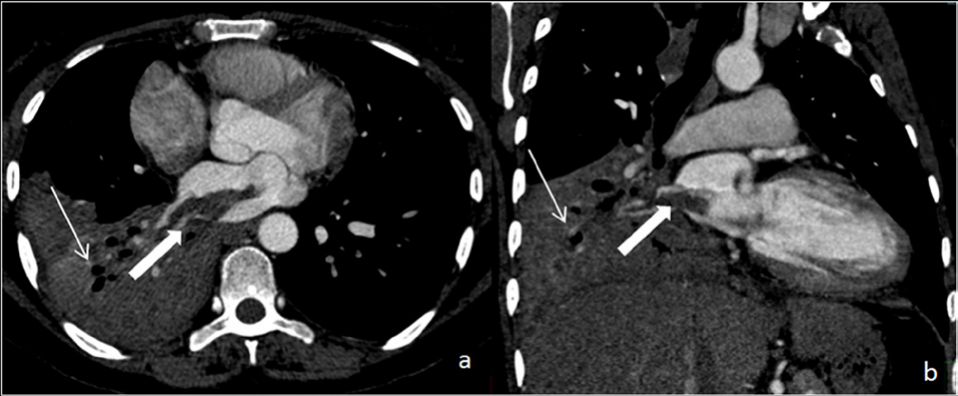
(a, b) Axial and oblique coronal CECT images showing thrombus in right inferior pulmonary vein extending into left atrial cavity (block arrows). Note the right lower lobe consolidation (straight arrows).

MDCT chest showed consolidation with underlying cavitation of right lower lobe (Figure 1a). Extensive discrete and confluent lung nodules were noted bilaterally with tree in bud appearances (Figure 1b). There was thrombosis in right inferior pulmonary vein extending into left atrial cavity reaching upto the mitral valve (Figure 2). Other findings included splenomegaly and splenic infarcts.

Possible infection related thrombosis was thought and patient was started on anticoagulation. No evidence of endobronchial tuberculosis was noted bronchoscopically. Gene X-pert on bronchoalveolar lavage fluid was positive for myocobacterium TB and Rifampicin resistance was not detected. Patient was continued on category 2 ATT (daily regimen) along with IV antibiotics and supportive measures. Patient made a gradual improvement with treatment. Adequate anti-coagulation was achieved and was being discharged in a stable condition.

## Discussion

Pulmonary TB is one of the most prevalent chronic infectious disease in India and worldwide. The mortality rate in pulmonary tuberculosis is high in those with advanced disease and in presence of complications. TB can present with wide variety of manifestations of which Haematological complications are rare to occur. There are many case reports on deep venous thrombosis in literature. There were 30 cases of pulmonary TB with DVT were reported after a retrospective study for 3 years.^5^ Other than extremity venous thrombosis, other vein thrombosis were also reported. A case of abdominal TB and portal vein thrombosis in young male patient was reported by Ozsekar et al.^6^ Gogna et al. reported a case of hepatic vein thrombosis in TB.^7^ TB meningitis with superior sagittal vein thrombosis was also reported.^8^ Vascular complications may even progress to the extent of pulmonary thromboembolism.^9^ Central retinal vein obstruction may also be a presenting feature in pulmonary tuberculosis.^10^ Case of tubercular lung cavitation with emboli in bilateral proximal pulmonary artery was reported.^11^ Vascular complications often correlate with the severity of tuberculosis.^12^

Vascular complications in TB are multifactorial. Activation of monocytes, macrophages there by release of interleukins and cytokines causing endothelial injury has been implicated. Increase in fibrinogen, impaired fibrinolysis, decrease in antithrombin III and reactive thrombocytosis can predispose to venous thrombosis in TB.^13^

In our case, MDCT showed consolidation with underlying cavitation of right lower lobe and extensive lung nodules bilaterally with tree in bud appearances. Also there was pulmonary vein thrombosis with extension into left atrium. Our case is unique in the perspective of TB with pulmonary venous thrombosis and left atrial extension which we believe is the first case reported in literature.

## Conclusion

Pulmonary TB is one of the most prevalent chronic infectious disease in India and worldwide. The mortality rate in pulmonary tuberculosis is high in those with advanced disease and in presence of complications. Our case emphasizes the complication of pulmonary venous thrombosis with atrial extension in patients with pulmonary TB. There is need to establish an early diagnosis and institution of prompt treatment with anti-coagulation while continuing ATT.

## Competing interests

The authors have no conflict of interest.

## Ethical approval

Patient data was anonymized and informed consent of the patient was taken.

## References

[1] Kumarihamy KW, Ralapanawa DM, Jayalath WA (2015). A rare complication of pulmonary tuberculosis: a case report. BMC Res Notes.

[2] Ambrosetti M, Ferrarese M, Codecasa L, Besozzi G, Sarassi A, Viggiani P (2006). Incidence of venous thromboembolism in tuberculosis patients. Respiration.

[3] Gogna A, Pradhan GR, Sinha RS, Gupta B (1999). Tuberculosis presenting as deep venous thrombosis. Postgrad Med J.

[4] Sharma RR, Acharya KV, Poornima V (2007). A rare complication of pulmonary tuberculosis - case report. JIACM.

[5] Kouismi H, Laine M, Bourkadi JE, Iraqi G (2013). Association of deep vein thrombosis with pulmonary tuberculosis. Egypt J Chest Dis Tuberc.

[6] Ozseker B, Ozseker HS, Kav T, Shorbagi A, Karakoc D, Bayraktar Y (2012). Abdominal tuberculosis leading to portal vein thrombosis, mimicking peritoneal carcinomatosis and liver cirrhosis. Acta Clin Belg.

[7] Gogna A, Grover S, Arun A, Saluja S (2004). Isolated hepatic inferior vena cava thrombosis in a case of tuberculosis. JIACM.

[8] Sundaram PK, Sayed F (2007). Superior sagittal sinus thrombosis caused by calvarial tuberculosis. Neurosurgery.

[9] Goncalves IM, Alves DC, Carvalho A, do Ceu Brito M, Calvario F, Duarte R (2009). Tuberculosis and Venous Thromboembolism: a case series. Cases Journal.

[10] Fullerton DG, Shrivastava A, Munavvar M, Jain S, Howells J, MacDowall P (2007). Pulmonary tuberculosis presenting with central retinal vein occlusion. Br J Ophthalmol.

[11] Ekukwe NC, Bain LE, Jingi AM, Sylvia K, Mintom P, Menanga A (2014). Bilateral pulmonary embolism in a patient with pulmonary tuberculosis: a rare association in Yaoundé, Cameroon. Pan Afr Med J.

[12] Ortega S, Vizcairo A, Aguirre IB, Sanchez PM, Saade MEA, Galan EA (1993). Tuberculosis as risk factor for venous thrombosis. Ann Med Intern.

[13] Robson SC, White NW, Aronson I, Woolgar R, Goodman H, Jacobs P (1996). Acute-phase response and the hypercoagulable state in pulmonary tuberculosis. Br J Haematol.

